# Multiple sclerosis and self-alienation: a study based on self and others representations

**DOI:** 10.1186/s40359-024-02264-w

**Published:** 2024-12-18

**Authors:** Leila Ziaie, Mohammad Ali Mazaheri, Abbas Zabihzadeh, Masoud Etemadifar, Omid Shokri, Richard J. Contrada

**Affiliations:** 1 Department of Psychology, Faculty of Education & Psychology, Shahid Beheshti University, District 1, Evin, Daneshjou Boulevard, Tehran, 198396411 Iran; 2 Department of Neurosurgery, School of Medicine, Isfahan University of Medical Science, Isfahan, Iran; 3 Rutgers, The State University of NJ, Piscataway, NJ 08854 USA

**Keywords:** Comfortable Interpersonal Distance (CID), Mental representations, Multiple sclerosis (MS), Others, Self

## Abstract

**Background:**

Multiple sclerosis (MS) is an autoimmune disease of the central nervous system. MS causes many changes in the lives of its patients, forcing them to renegotiate their lives. Part of these changes are related to patients’ self- and others- mental representations. Despite the importance of mental representations in changes caused by or adaptation to MS, limited research has been conducted to examine the mental representations of people with MS.

**Methods:**

This study examines the mental representations that Healthy Controls (HC) and people with MS (PWMS) have of themselves and others, including childhood and current self-images, as well as those of their mothers, love partners, and close friends. In both groups (HC (*n* = 82) and PWMS (*n* = 82)), participants completed demographic variables as well as a modified version of the comfortable interpersonal distance (CID) task in both passive (when protagonists approached them) and active modes (when participants approach protagonists). Participants estimated the point at which they felt discomforted.

**Results:**

The PWMS group preferred a significantly larger interpersonal distance than the HC group for both current self-image and love partner. There is no difference between the two groups in preferring interpersonal distance from their childhood self-image, mother, and friends.

**Conclusions:**

Based on the research results, it seems that PWMS experience a kind of alienation at the level of self-mental representations. This research can be helpful in modifying MS interventions and increasing the engagement of support systems.

## Introduction

Multiple sclerosis (MS) is a chronic autoimmune inflammatory disease that damages the myelin in the central nervous system (CNS) [1]. MS is characterized by a range of symptoms that affect various functional systems. Common symptoms of MS include cognitive dysfunction, fatigue, depression, motor weakness, diplopia, visual loss, sensory symptoms, bladder and bowel dysfunction, spasticity, tremor, and pain. Psychiatric symptoms, such as depressive, bipolar, anxiety, schizophrenic, and obsessive-compulsive syndromes, are frequent in People with MS (PWMS) and significantly impact the quality of life [2–5].

MS, as a chronic illness, does not only affect the body [6]. It seems that the wide range of symptoms of MS, along with its high prevalence in the ages of 20 to 40 [7, 8], which is known as the period of self-development [9] and transitioning into adult roles such as intimate relationships, parenting, and work roles, affects the formation of a person’s sense of self, body, and performance leading to feelings of loss of self and changing identity [10–12]. MS can have a negative psychological effect on patients, and they need to redesign their lifestyle, adjust their time, and cope with the uncertain path of the disease [12]. Therefore, MS forces people to have a fundamental rethinking and re-evaluation of previous biographies and mental representation of themselves, expectations, pursuits, and relationships no longer comport with the new realities and limitations of being diagnosed with MS and to think about their current self-concept and the ideas they have about their future [13, 14]. These ruptures have been conceptualized as “biographical disruption” for patients [15], which can damage self-representation [16], derailing all aspects of life trajectory. Biographical disruption can involve a loss of continuity, coherence, and control over a patient’s life story, as well as a loss of various domains of one’s biography, such as physical abilities, appearance, emotions, cognition, personality, relationships, work, hobbies, plans, routines, expectations, and goals [6, 17, 18].

Multiple studies have investigated the impact of MS on patients’ lives, referred to as biographical disruption. For example, Individuals with MS tend to have lower overall self-concept [19, 20], negative attitudes toward their past and deterministic views of their lives [21]. Furthermore, MS can affect self-continuity and self-awareness in various ways. leading to a distorted perception of their functional abilities [22] and experience a dissociation between body ownership and self-location, indicating a disruption in bodily self-consciousness [10]. The clinical symptoms and unpredictability of MS can also impact patients’ self-image and self-appraisal, affecting their emotional status and behavior [21]. Furthermore, the desire to retain normalcy while managing the increasing disease burden can lead to a loss of self and a need for timely and contextual self-management strategies [23]. PWMS also reported impaired body image [24], worse body appraisal, and sexual problems with women more focused on physical appearance and men on sexual issues [23, 25]. MS can influence emotional functioning by causing difficulties in recognizing facial expressions and experiencing emotions [26] predicting poorer social functioning [27]. There are often higher levels of work difficulties and adverse outcomes for individuals with MS, such as the termination of their employment and a decrease in work participation, such as voluntary and involuntary [28]. The effects of MS on relationships include challenges in daily activities, emotional well-being, and maintaining relationships [29]. Also, MS affects interpersonal relationships by changing the dynamics and roles within the relationship [11] and experiencing guilt and blame towards PWMS or their partners [12].

Although MS is a potential threat, the impact of MS disease on patients depends on various factors such as the individual’s embodiment and physical ability to engage with daily life [30], presence of mood and mental health disorders [31], social support [32] and how to interpret and process threats related to physical health based on their mental representations [33]. Therefore, although the experience of chronic diseases may lead a patient to a changed self and others’ mental representations, giving meaning to the experience of chronic disease – and, as a result, experiencing a kind of disruption in the biography, identity, and mental representations - is itself influenced and formulated by the mental representations.

The concept of mental representations refers to the organization of conscious and unconscious cognitive, emotional, and experiential components of early interpersonal experiences with significant others. It represents a person’s developmental level and various aspects of his mental life, such as impulses, emotions, instincts, and fantasies [34]. Mental representations are seen as a tool for understanding and adapting to the environment. They also shape how people perceive and respond to their experiences, particularly threatening ones [33]. Mental representations can influence behavior in different situations in different ways. Still, two methods are emphasized the most. The first influence of mental representations is their bias in accessing or interpreting information. A defensive exclusion model and a schema-congruent model are two models of biased information processing based on attachment theory. According to the defensive exclusion model, individuals are unconsciously motivated to restrict access to disturbing or threatening information by engaging in defensive strategies such as attention avoidance, blunting, suppression, and counterarguing [35]. These defensive processes have been studied in various domains, including colorectal cancer screening [36]. Schema-congruent models, on the other hand, tend to attend to and process information that aligns with their preexisting mental representations, while incompatible information tends to be ignored. Second, their content biases in how others can be perceived, self-representations, mind-body relations, relationships, social attributions, and other understanding domains. These biases occur regarding early interactions with significant others [37]. Mental representations that include accurate beliefs about self, others, and the world, meaningful goals, and effective strategies to accomplish those goals contribute to a sense of coherence and stability, leading to better psychological and physical health [38]. On the other hand, distorted or underdeveloped mental representations can lead to maladjustment, emotional pain, poor physical health, self-defeating responses, stress, anxiety, depression, and poor motivation [39, 40]. Therefore, the development and structure of a person’s mental representations play a crucial role in their overall well-being, including their physical health [41]. In summary, in facing experiences that threaten the continuity and integrity of oneself and daily functioning, including physical or mental illnesses, mental representations by both process and content can significantly affect how a person perceives, interprets, and responds to them.

Although primary relationships with significant others form mental representations that are almost constant and stable, mental representations are dynamic and can be updated, elaborated, or replaced as life circumstances change [42]. Social relationships, the passage of time, and life experiences can influence changes in mental representations. Major life experiences such as parents’ death, divorce, and illness can have negative effects, fluctuations, or changes in mental representations. On the other hand, interacting with significant others, such as a partner, friend, or therapist, can lead to mental representations in a positive direction [43].

Mental representation has been evaluated using different scales due to its importance in personality organization. Most of these scales have emphasized the implicit aspects of mental representations (for example, Blatt’s Differentiation-Relatedness Scale and Blatt’s Qualitative-Thematic Scales [34]). The attachment approach uses self-report scales and attachment interviews to assess mental representations (internal work models) in adulthood. The comfortable interpersonal distance (CID) test measures individuals’ mental representations based on the distance participants maintain with images of different characters. The emotions created in an interaction [44], as well as the perceived threat from another [45], can influence adopting distance so the preferred distance can reflect some aspect of mental representation. Accordingly, the distance from the individual’s mental representations of characters can reveal the quality of the mental representation. The participants’ degree of intimacy, acceptance, and emotion experienced while interacting with the characters is reflected in the distance they adopt towards each character during the CID task. Therefore, the narrower the space is, the more pleasant the mental representation of the individual interacting will be.

The CID test was employed to measure mental representations in various populations, including borderline personality disorder (BPD) [46], depression, and schizophrenia patients [47]. For these clinical populations, self- and others-mental representations are disturbed and negative to the point where confronting oneself or significant others (such as family members, love partners, and close friends) results in discomfort. Therefore, adopting a greater interpersonal distance is part of the ego-oriented defense against negative effects and emotional distress [47].

### Current study

Extensive literature based on qualitative narrative analysis shows how MS affects patients’ lives (e.g., [48]). However, limited quantitative studies have been conducted to assess self- and others’ mental representations in PWMS. The present study evaluates patients’ mental representation of themselves and others close to them with the comfortable interpersonal distance test. Accordingly, the hypothesis includes the following: First, regarding the impact of early interactions with significant others on forming self-mental representation, we predict that PWMS would maintain more interpersonal distance from the mother -as an attachment figure- relative to the HC group. Secondly, due to the impact of MS on close relationships, we expect that PWMS will exhibit an increased interpersonal distance from their love partner and close friend compared with non-diseased individuals. Our final and most crucial hypothesis is that because of biographical disruption and the importance of self-mental representation in experiencing it, we hypothesized that PWMS would choose a larger interpersonal distance from their current self-image and childhood self-image than healthy participants due to the impact of mental representation on the perception of biographical disruption.

## Methods

### Participants

In this study, 82 adults with MS from the Isfahan MS Association participated by convenience sampling method. Participants were selected based on their availability and willingness to participate, rather than through random sampling. These patients include 25 males and 57 females with an average age of 35 (± 6.74). The interquartile range (IQR) of the Expanded Disability Status Scale (EDSS) was 1.5 (Q1 = 1, Q3 = 3). All participants were diagnosed with MS by a neurologist at least one year prior to the study. Exclusion criteria for PWMS are as follows: (1) Currently or previously diagnosed with psychotic disorders, and/or (2) any developmental disorders (e.g., autism spectrum disorder), and/or (3) severe head injury or neurological diseases other than MS, and/or (4) EDSS score equal to 6 or higher. The EDSS is a standard assessment scale used by clinicians to quantify disability in PWMS. This scale is used to track disease course and evaluate the effectiveness of therapeutic interventions. The EDSS ordinal rating system uses 0.5-point increments to go from 0 (normal neurological condition) to 10 (death from MS). Higher scores indicate more severe disability, with scores of 6 and above representing significant disability. This study limited participants with MS to those with an EDSS score between 0 and 5.5 to avoid interference from disabilities in the results.

Eighty-two people from Isfahan City, Iran, with similar demographic characteristics to PWMS, participated as a control group (HC) by convenience sampling method, with an average age of 34.21 (± 8.07) (Appendix). After individuals with MS completed the task, their demographic data were collected. Subsequently, through a call for participants published on social media, healthy individuals with demographic characteristics similar to the MS group were invited to take part in the study. None of the participants in the HC had evidence of MS or other autoimmune diseases. In addition, all participants met the criteria for entering the research: all were (1) at least 20 and at most 50 years old, (2) understanding the experimental procedure and were able to follow it, and (3) having normal visual and auditory abilities. We received approval for the research procedure [ the name of the ethics committee moved for double-blind peer review]. Informed consent was provided by all participants. All participants entered the study voluntarily, and none withdrew after receiving explanations, during the task, or following its completion.

### Modified version of Comfortable Interpersonal Distance (CID)

Duke & Nowicki [49] introduced the comfortable interpersonal distance (CID) test to measure the comfortable distance between self and others in a paper and pencil format. Further studies have reported the validity and reliability of this test in different cultures. Additionally, the CID scores correlate highly with the optimal interpersonal distance preferred in daily actual face-to-face interactions [50]. The CID tests have previously been used to assess the mental representations of individuals and groups [46, 47]. In the present study, we used the CID task to measure the optimal distance from different personalities in the PWMS and the HC groups and the subsequent mental representations. In line with Perry et al. [51], we used a modified computerized task version. A similar version was used in previous related research (e.g [50]., and included two modes, according to Abdevali et al. [44, 46]. The version used in this study has also been previously applied in other Iranian research and found to be appropriate [46, 50].

**Fig. 1:**
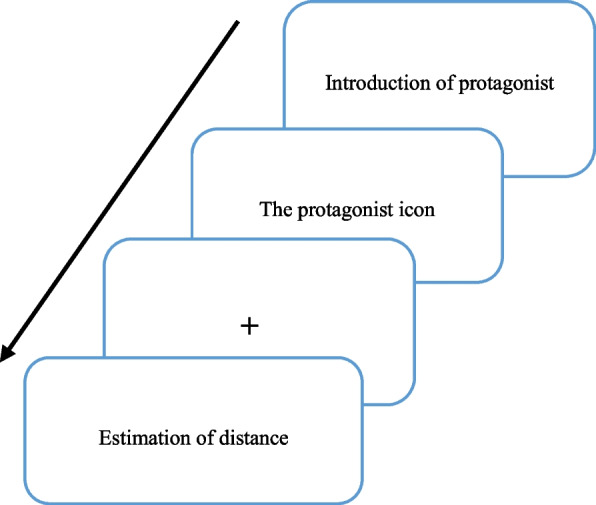
The order of each trial conducted in the modified version of the CID task in this study

During the passive mode, a participant’s icon is placed in the center of the room (circle) in each trial. After opening each door, the protagonist moves towards the icon to the extent the participant permits by pressing the space button. During the active mode, in each trial, the protagonist icon is placed in the center of the room, and a participant’s icon enters the circle from one of four doors and starts approaching the protagonist as far as he/she prefers (Fig. 1).

**Fig. 2:**
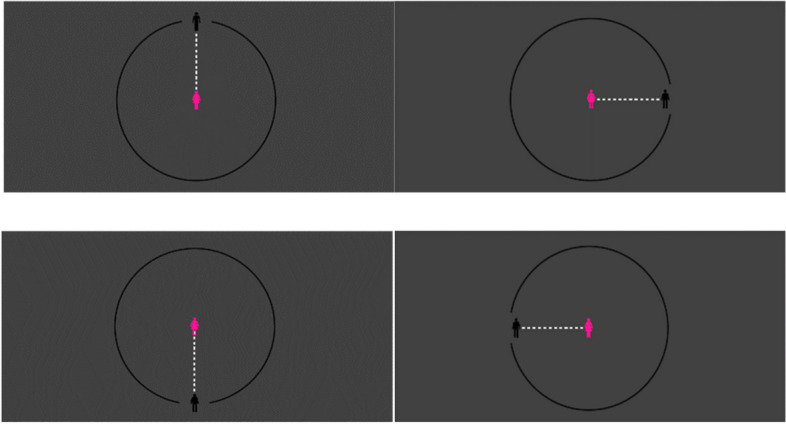
Images of four doors at 0, 90, 180, and 270 degrees in the modified version of the CID

We used the Unity software package to conduct the simulation. A 90 mm radius circle on the screen was displayed with 12 mm human icons in the center (Fig. 2). In both versions of the task, participants measured their optimal interpersonal distance toward each protagonist four times from one of the four to shorten the task time. For each protagonist, the mean distance was considered a comfortable interpersonal distance. Each participant completed 20 trials in each version, a total of 40. In the present study, we attempted to measure the comfortable interpersonal distance and mental representations of PWMS towards five protagonists. During the passive mode, participants were first presented with the approaching protagonist’s name (i.e., the icon representing themselves located in the center of the 15-in. computer screen) for 3s, followed by a fixation point (the plus sign) for 0.5s, then the icon of the protagonist approaching from a door. Also, in the active mode (when the participant icon approaches the protagonists), the participants estimated the distance they considered comfortable with them.

### Statistical analysis

As a preliminary step to conducting the parametric tests for comparing the two groups, Kolmogorov-Smirnov tests were conducted to assess normality and Levene’s tests for homogeneity of variance for CID task scores. To analyze the mean differences between the two groups in the CID task, we used the repeated measures ANOVA test since participants’ scores were analyzed based on their group (a between-subject variable) as well as in two experimental modes (passive and active) and for five protagonists (as two within-subject variables). In addition, Bonferroni’s post hoc test was also applied to compare two groups in the mean estimation of interpersonal distance from different protagonists.

## Results

The means and standard deviations of the comfortable interpersonal distances for the different protagonists in both active and passive modes are shown in Table 1.

**Table 1:** Mean and standard deviation of the optimal interpersonal distance from different protagonists in both active and passive modes

Protagonists	Groups	Passive mode		Active mode	
		Mean	SD	Mean	SD
Childhood self-image	PWMS	33.348	2.691	36.631	2.920
	HC	30.305	2.243	31.085	2.500
Current self-image	PWMS	33.929	2.649	37.028	2.724
	HC	27.547	2.148	30.488	2.406
Mother	PWMS	27.815	2.414	27.688	2.367
	HC	27.220	1.787	30.942	2.151
Love partner	PWMS	30.188	2.512	31.281	2.425
	HC	21.233	1.627	23.370	2.037
Close friend	PWMS	43.246	1.905	48.884	1.950
	HC	41.581	1.703	42.969	1.972

*PWMS *People with MS (*n* = 82), *HC *Healthy Controls (*n* = 82), *SD *Standard Deviation

We conducted 2 (groups: PWMS and HC) × 2 (experimental modes: passive and active) × 5 (protagonists: childhood self-image, current self-image, mother, love partner, and close friend) repeated measures ANOVA. There was a main effect for experimental modes [*F*(1,162) = 7.822, *p <* 0.05, ƞ2 = 0.046]. Also, there were significant main effects for protagonists [*F*(3.024,519.033) = 33.826, *p <* 0.05, ƞ2 = 0.173] and groups [*F*(1,162) = 4.516, *p <* 0.05, ƞ2 = 0.027]. Although the two-way interactions were not significant for experimental modes × groups [*F* (1,162) = 0.056, *p* = 0.814, ƞ2 = 0.000] and experimental modes × protagonists [*F*(3.647,590.831) = 0.496, *p* = 0.721, ƞ2 = 0.003], the interaction between protagonists and groups was significant: [*F*(4,519.033) = 2.412, *p <* 0.05, ƞ2 = 0.015]. Moreover three-way interaction was not significant [*F*(4,590.831) = 1.788, *p* = 0.129, ƞ2 = 0.011] (Appendix B). It is worth noting that we also conducted a 2 (groups: PWMS and HC) × 2 (gender: male and female) × 2 (experimental modes: passive and active) × 5 (protagonists: childhood self-image, current self-image, mother, love partner, and close friend) repeated measures ANOVA. There were no significant main effects for gender [*F*(1,160) = 3.711, *p =* 0.06] or interactions between gender and group [*F*(1,160) = 1.024, *p =* 0.313]. Therefore, to avoid complexity, we focused on a 2 × 2 × 5 repeated measures ANOVA and did not include gender.

As shown in Table 2, the Bonferroni post hoc test was used to compare groups based on the means estimated interpersonal distance from different protagonists. Compared to the HC group, the PWMS group preferred a larger interpersonal distance from their current self-image and love partner. Although the comfortable interpersonal distance from the mother was larger in the HC group than in the PWMS group, this difference was insignificant (*p* = 0.631). Furthermore, the PWMS group preferred larger comfortable interpersonal distance from their childhood image and close friends, but this difference was not significant either (*p* = 0.204, *p* = 0.120).

**Table 2:** Results of Bonferroni post hoc test for mean difference between PWMS and HC groups for each protagonist

Protagonists	Mean difference	Standard errors	Sig.
Childhood self-image	4.294	3.369	0.204
Current self-image	6.461	3.218	0.046
Mother	−1.330	2.762	0.631
Love partner	8.433	2.792	0.003
Friend	3.790	2.427	0.120

*PWMS *People with MS (*n* = 82), *HC *Healthy Controls (*n* = 82)

**Fig. 3:**
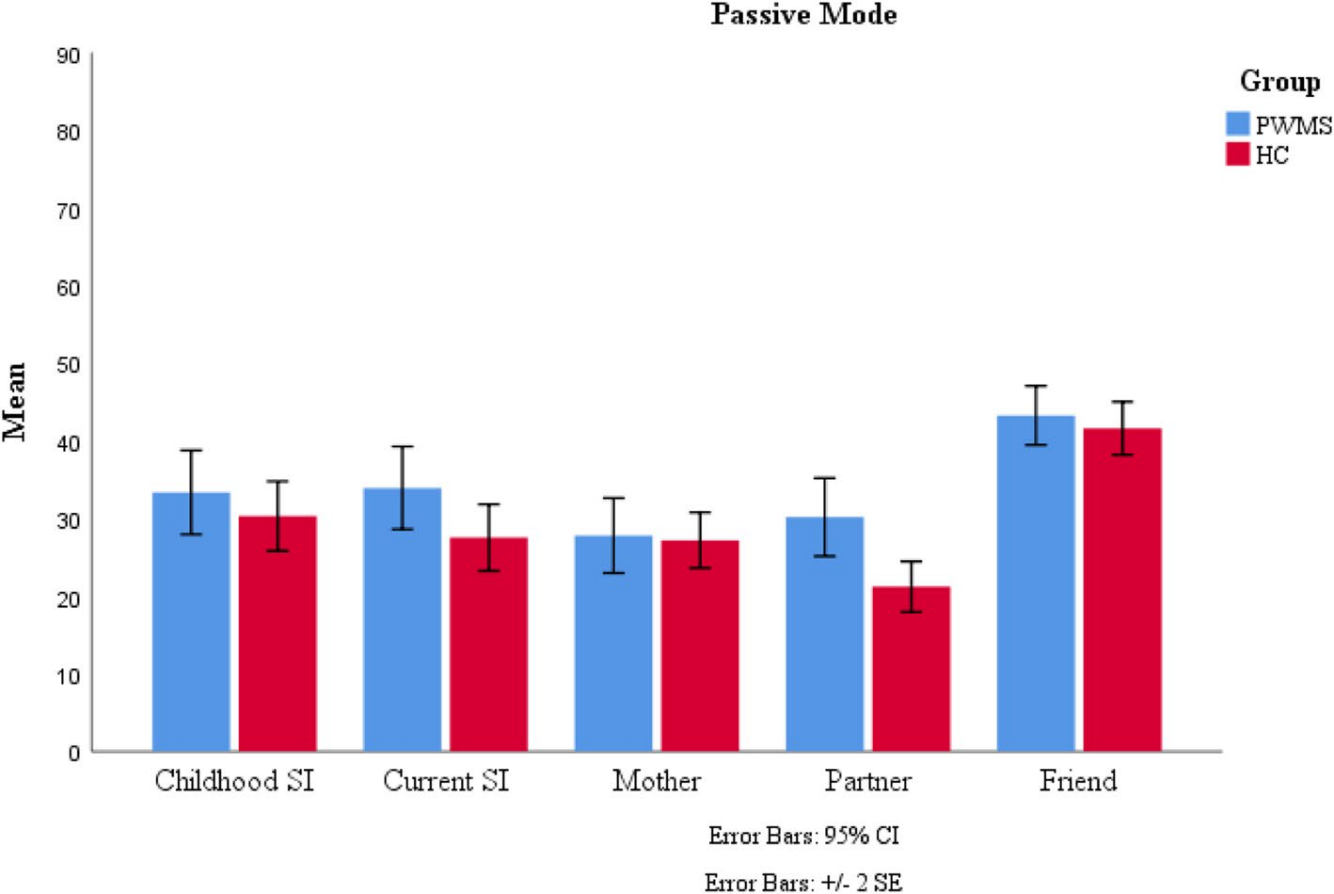
Mean of the comfortable interpersonal distance from different protagonists in two groups and passive experimental mode. *Note*. HC=healthy controls, PWMS= patients with multiple sclerosis

**Fig. 4:**
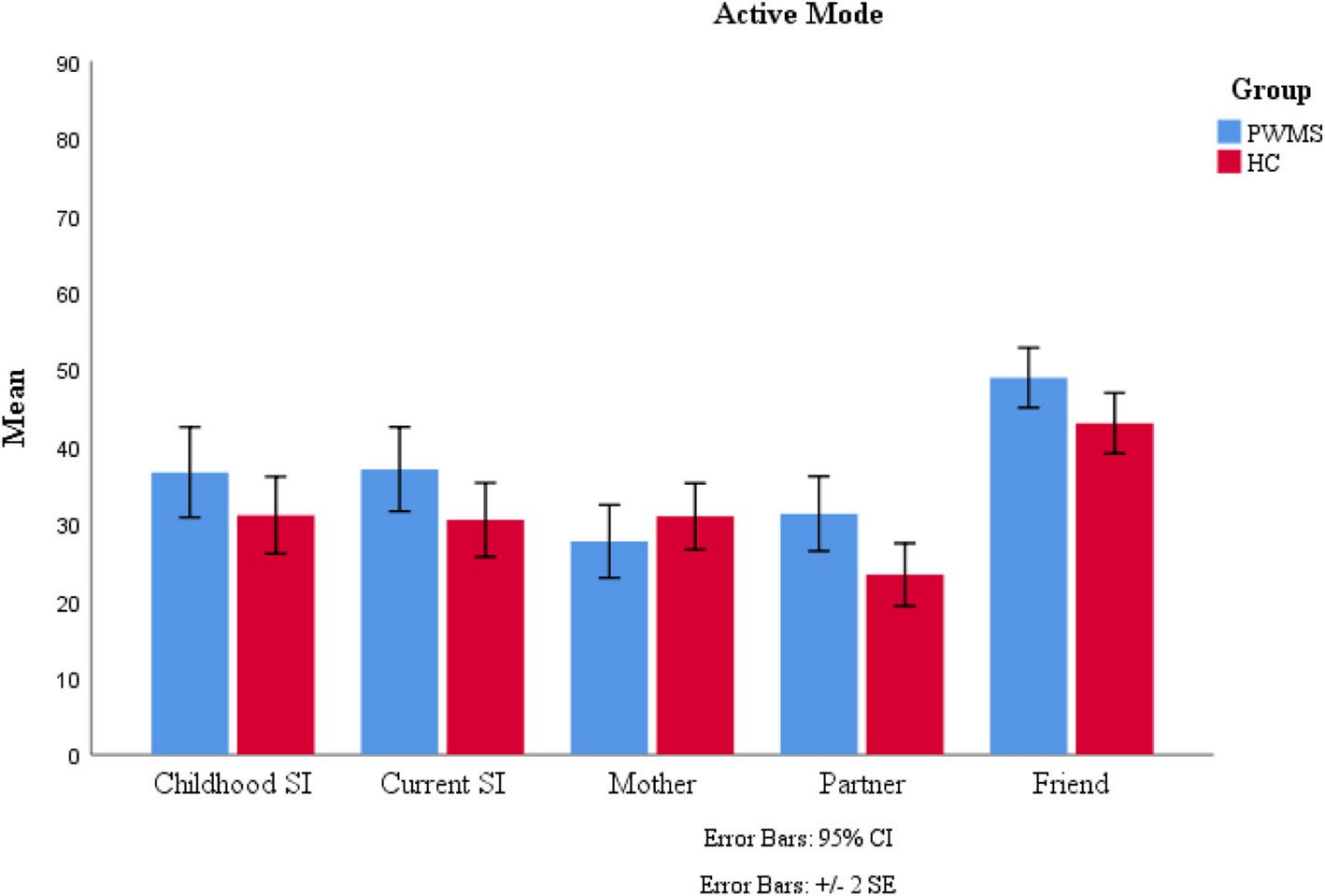
Mean of the comfortable interpersonal distance from different protagonists in two groups and active experimental mode. *Note*. HC=healthy controls, PWMS= patients with multiple sclerosis

Figures 3 and 4 illustrate the comfortable interpersonal distance from different protagonists in the PWMS group compared to the HC group in the passive and active experimental modes, respectively.

## Discussion

The study aimed to evaluate mental self- and others- -representations in PWMS. While several papers have acknowledged the impact of MS on lived experience, limited quantitative studies have been conducted to examine the effects of MS disease on mental self and others’ representations implicitly. Comfortable interpersonal distance task (CID) was used to evaluate mental self- and others- -representations in PWMS and HC. According to our findings, there are significant differences in the mental representations between PWMS and HC. The PWMS group preferred a larger distance from the protagonists of their current self-image and their love partners but not their childhood self-image, mother, and close friend compared to the HC group.

Contrary to our first hypothesis, the preference for the distance between PWMS and HC does not differ when faced with the image of their mother (as an attachment figure). In most cultures, the mother is considered the child’s primary caregiver. Therefore, interactions and attachment with the mother are fundamental in forming the structure and content of mental representations, especially mental self-representations. Mental self-representations are initially a part of others’ mental representations, which become entirely distinct during the first two years of life [52]. Therefore, other mental representations, particularly those of the mothers, provide the foundation for mental self-representation. Thus, the image of the mother is often considered the closest concept to self-representation. Understanding the mental representations of PWMS mothers helps identify how MS biographical disruption is processed. Based on the research results, the emotions experienced in facing the images of mothers in PWMS are not different from those in HC. Considering that the mental representations of the mother were formed before MS diagnosis, the quality of the relationship of PWMS with their mother is not different from that of non-patients. A positive relationship exists between attachment to parents and global satisfaction in adulthood [9]. Also, secure attachment to parents is associated with positive coping strategies in adulthood [53, 54], which is necessary to adapt to MS.

Consistent with the second hypothesis, PWMS tend to maintain a greater interpersonal distance from their love partner. As a chronic disease, MS affects patients’ intimate relationships. Previous studies have demonstrated that MS affects intimate relationships by increasing intimacy or disrupting it [11]. As the results of the present study show, PWMS experience more negative emotions in their intimate relationships with their love partners, so MS disease often has a negative effect on intimate relationships. This issue can be due to sexual dysfunction [55] and emotional closeness [11] with the love partner, as well as the increase in irritability towards them [11, 12]. PWMS also feel guilty about themselves or their partners [12]. They may feel guilty for their sexual dysfunction [56]. They may also be ashamed of their emotional partner due to illness and physical condition [48]. Despite the high acceptance of the love partner of PWMS, patients may believe they are a burden on their partner. In addition, their love partner may question their symptoms, think they are exaggerating their symptoms, or accuse them of being lazy or selfish. All these factors result in a strong negative perception of their relationship with their spouse or partner [11]. Therefore, they maintain a greater distance from the mental representation of their partners.

Distancing from others can be viewed as a passive defense mechanism that allows individuals to maintain a secure zone to protect themselves from external and internal threats [57]. The emotion created when facing another [44], the perception of threat [45], and shame [58] in close proximity to others are critical factors in regulating interpersonal distance. Thus, it can be concluded that PWMS feel threatened and/ or shamed when confronted with their love partners’ images by bringing up mental representations of them and seeking to avoid negative emotions. As a result, they prefer more interpersonal distance.

However, contrary to our expectation, there is no difference between the two groups’ predilection for interpersonal distance when confronted with a close friend. This finding means that PWMS do not experience negative emotions such as threats or shame in their friendships more than the HC group. MS disease forces people to organize a new life and create new social relationships, usually with the treatment staff and other PWMS, which may evoke fewer negative emotions and thus not prefer greater interpersonal distance. It should be noted that the friends in this study were not determined whether they were sick. Regardless, having a positive mental representation of friends can represent the patients’ positive social connections and act as a source of support for them. Friendships benefit people with disabilities, including those with multiple sclerosis (MS). Friendships offer physical and emotional support and enjoyment, which is essential to adopting MS [59]. They may also provide support for self-esteem and encourage PWMS to cope with MS in various ways [60]. Additionally, friendships positively impact subjective well-being and can protect against mental health problems such as anxiety in PWMS [32, 61]. They provide companionship, emotional and practical support, and a sense of belonging [62].

Consistent with the final hypothesis, PWMS adopted greater interpersonal distance from their current self-image but not from their childhood self-image. As can be seen from the research results, PWMS mental self-representations, in comparison with HC, provoke more negative emotions that can be linked to the content of mental representations of the self. One of the significant aspects of biography disruption in PWMS is related to self-concept and self-definition [20, 22]. Self-concept of PWMS was predominately experienced negatively. PWMS describe themselves as dependent, useless, insufficient, unpredictable, and untrustworthy when explaining how MS has affected their perceptions of themselves [13]. Therefore, PWMS usually experience mental self-representations with negative emotions such as anger, threat, or shame and distance themselves from their current self-image to regulate their emotions. It is worth mentioning that although the biography disruption may cause a change in the mental representations and emotions affecting it because the experience of the biography disruption itself is influenced by the mental representations, especially mental representations of self and significant others (attachment figures), there is a possibility that the structures of mental representations of self, especially current self-image, were disturbed before the onset of MS.

Based on the study’s results, it appears that PWMS have problems with the self and its concept (especially the current self-mental representation). The negative emotion experienced when interacting with one’s current self-image. As a result, distance adoption among PWMS is similar to those found for people with negative self-concepts [44] and people with depression [47]. According to Ponizovsky et al. [47], self-alienation and internal disgust are part of the ego-oriented defense strategies of these people to avoid experiencing negative effects and emotional distress associated with their psychopathology.

In adulthood, the most significant source of an individual’s attachment is their love partner, which is vital to life satisfaction. PWMS need the acceptance and support of their partners, which makes this source of attachment more essential for them. As the results showed, PWMS experience negative emotions in their relationships with their partners, which decreases their life satisfaction.

Progressive decline in executive functions associated with frontal lobe impairments, including deficits in attention, memory, and decision-making, can significantly influence how PWMS processes information and applies defensive strategies as introduced by the defensive exclusion model. For example, impairments in executive function may cause certain defensive responses, such as attention avoidance or information suppression, in the face of threatening health information. As MS progresses, brain volume loss and associated cognitive deficits may alter how individuals mentally represent their condition and cope with health-related stressors. Future research might look at how the level of cognitive impairment in PWMS affects processing of health threaten information and if distinct cognitive deficiencies impact the choice or effectiveness of defensive strategies.

In practice, these outcomes could direct the development of MS care interventions that emphasize restoring one’s self-representation and developing ties with significant others. For example, self-compassion exercises or cognitive behavioural techniques might be used in therapy interventions to help PWMS feel less alienated from themselves and more accepted. These interventions could foster a more cohesive self-representation and support patients in processing alterations they may experience. Interventions that focus on couples relationship dynamics, may also boost the support system and lessen the feelings of being a burden that many PWMS encounter. For instance, couple-based therapy may address how to communicate about MS symptoms and encourage mutual support and understanding, eventually enhancing interpersonal relationships.

Based on the evidence that PWMS prefer greater interpersonal distance with their current self-image and significant others, we hypothesize that interventions designed to improve self-representation and relationship dynamics may not only enhance psychological well-being but also reduce interpersonal distance preferences. Future research could examin whether these intervention can improve self and other representation, which could lead to greater comfortable interpersonal distance and decrease in social isolation.

These findings must be considered in light of the study’s strengths and limitations. First, this study did not account for potential variations in visual function, motor coordination, reaction time, or attention capacities (including focused, sustained, and divided attention), which are commonly affected in PWMS and may impact task performance. Additionally, factors such as the degree of brain atrophy and the duration of MS since symptom onset were not examined. These variables could influence participants’ cognitive and physical capacities in the passive and active modes of the task. Future studies that address these factors may yield further insights into their potential impact on the results.

Second, the research was conducted during the Covid-19 pandemic. Social distancing strategies, crucial to preventing COVID-19, have also affected participants regulating interpersonal distance. It can limit the generalizability of the results at other times. Also, limiting the PWMS group to a specific EDSS score (equal to or less than six), as well as factors such as the duration of the disease, whether symptoms were present at the time of the study, and the medications used, may influence the results. Further research on these factors may contribute to a better understanding of the issue in the future.

Finally, differentials with subgroups of MS, distinct patterns of progression, relapse frequency, and baseline disability, along with factors such as the efficacy, tolerability, and side effects of disease-modifying therapies, disease stability, and recent relapse treatments (e.g., steroid use), may all influence self- and other representations. Since these clinical factors were not controlled in this study, generalizing the results to larger groups of MS patients may be challenging. Further research on these factors may contribute to a better understanding of the mental representations of PWMS in future.

## Conclusions

In conclusion, these results reveal that the self-mental representation of PWMS is distorted. An individual’s self-mental representation is the closest psychological concept to himself. Keeping distance from self-image more than others can indicate biographical disruption related to multiple sclerosis. Although the childhood mental self-representations and mental representation of the attachment figure (mother) were not different in the two groups of this research, considering the possibility of disruption in the structures of mental self-representation before morbidity with MS, attention should be paid to psychological mechanisms in MS progressive as well as the psychological facilitators of its development. It is especially crucial to recognize the impact of the first experiences with significant others on developing mental self-representations and significant others and the relationship between the mind and body. From this point of view, it is worthwhile to conduct more studies on the content of mental self-representations and mental representations of significant others to identify the signs of “alien self” and “self-alienation” in PWMS, especially in the years before the onset of the disease. It is determining whether or not self-attack is a psychological translation of MS - one of its signature symptoms - before the onset of illness.

## Data Availability

"The materials and data that support the findings of this study are available from the corresponding author upon reasonable request."

## Appendix

The difference between the PWMS and HC groups in demographic variables

**Table:**

Variable	Catergory	Group		Value	Sig.
		PWMS	HC	1.318	0.251
Sex	Male	25	32		
	Female	57	50		
Age	-	M = 35	M = 34.21	0.672	0.503
Marital status	Single	29	25	1.526	0.466
	In relationship	52	57		
	Other	1	0		
Education	Under Diploma	2	1	6.128	0.409
	Diploma	12	8		
	Associate Degree	9	4		
	Bachelor	42	44		
	Master	13	21		
	PhD	4	3		
	M.D	0	1		
Socioeconomic status	Lower	8	5	5.705	0.222
	Lower-middle	18	9		
	Middle	45	53		
	Upper-middle	11	14		
	Upper	0	1		

*PWMS *People with MS (*n*=82), *HC *Healthy Controls (*n*=82)

## Abbreviations

MS: Multiple sclerosis
PWMS: People with MS
HC: Healthy Controls
